# Supplementary material for: The Relation between Physiological Parameters and Colour Modifications in Text Background and Overlay during Reading in Children with and without Dyslexia
Source: Brain Sci. 2021 Apr 25;11(5):539. doi: 10.3390/brainsci11050539 (PMC8146078; doi:10.3390/brainsci11050539)
Supplement: Supplementary file 1 [file brainsci-11-00539-s001.zip › brainsci-1165078-supplementary.pdf]

Supplement Table S1. All background/overlay colours compared with white colour for each parameter in the groups (dyslexic with orange colour; non-dyslexic with green, $p<0.05$)

Colours	Red	Blue	Yellow	Orange	Purple	Turquoise	Red O	Turquoise O	Blue O	Orange O	Purple O	Yellow O
Reading duration (ms)			.048	.043				.005 / 0.17				
Fixation Count				.006						.047		
Fixation Frequency (count/s)		.051						.021				
Fixation Duration Total (ms)		.000		.025				.016				
Fixation Duration Average (ms)		.043						.025		.014		
Saccade Count		.019		.021			.021			.017		
Saccade Frequency (count/s)		.043 / .038					.035	.019 / .030		.050		.051
Saccade Duration Total (ms)		.035 / .048				.053				.035		
Saccade Duration Average (ms)												
Alpha (μV2)												
Beta (μV²)					.010	.029	.024	.023			.052	
Delta (μV²)												
Theta (μV²)								.003				
Whole range (μV²)												
EDA(uS)												
Mean RR (ms)	.043	.028				.013				.022		.033
STD RR (ms)												
Mean HR (beats/min)		.033				.015				.024		.032
STD HR (beats/min)												
RMSSD (ms)												
CVRR=SDRR/MeanRR (ms)												

* Wilcoxon Signed Ranks Test (Reading duration, Eye-tracking, EEG); * * *Paired Samples Test (EDA, HRV).*
